# Processing, Structural Characterization and Comparative Studies on Uniaxial Tensile Properties of a New Type of Porous Twisted Wire Material

**DOI:** 10.3390/ma8095266

**Published:** 2015-08-27

**Authors:** Fei Wu, Zhaoyao Zhou, Liuyang Duan, Zhiyu Xiao

**Affiliations:** School of Mechanical and Automotive Engineering, South China University of Technology, 381 Wushan Road, Tianhe District, Guangzhou 510640, China; E-Mails: w.f10@mail.scut.edu.cn (F.W.); me201410100179@mail.scut.edu.cn (L.D.); zhyxiao@scut.edu.cn (Z.X.)

**Keywords:** porous twisted wire materials, structural characterization, tensile failure, sintering parameter, porosity, wire diameter, sampling direction

## Abstract

A self-developed rotary multi-cutter device cuts stainless steel wire ropes into segments to fabricate twisted wires. Stainless steel porous twisted wire materials (PTWMs) with a spatial composite intertexture structure are produced by the compaction and subsequent vacuum solid-phase sintering of twisted wires. The stainless steel PTWMs show two types of typical uniaxial tensile failure modes, *i.e.*, a 45° angle fracture mode and an auxetic failure mode (the PTWMs expand along the direction perpendicular to the tension). The effects of the sintering parameters, porosities, wire diameters, and sampling direction on the tensile properties of the PTWMs are carefully investigated. By increasing the sintering temperature from 1130 °C to 1330 °C, the tensile strength of the PTWMs with 70% target porosity increased from 7.7 MPa to 28.6 MPa and the total failure goes down to 50%. When increasing the sintering time from 90 min to 150 min, the tensile strength increases from 12.4 MPa to 19.1 MPa and the total failure elongation drops to 78.6%. The tensile strength of the PTWMs increases from 28.9 MPa to 112.7 MPa with decreasing porosity from 69.5% to 46.0%, and the total failure elongation also increases from 14.8% to 40.7%. The tensile strength and the failure strain of the PTWMs with fine wires are higher than those of the PTWMs with coarse wires under the same porosity. Sampling direction has a small influence on the tensile properties of the PTWMs.

## 1. Introduction

Porous metal materials are currently attracting a large amount of research interest. They are applied to some critical applications such as heat transfer, energy absorption, protective devices, biomedical devices, catalyst support, filtration, acoustic damping, and others because of their porous structure and unique mechanical properties [1,2]. Neelakantan [1] defines porous materials fabricated by gathering many more fibers, wires or rods and bonding them at intersecting points as the “fiber networks”. Several previous experimental studies have been performed to explore the fabrication and mechanical response of metallic fiber networks. Fiber networks are sometimes classified according to whether they are periodic or stochastic. Stochastic metallic fiber networks primarily include entangled metallic wire materials or wire mesh, e.g., non-woven wire structures [3,4], quasi-ordered entangled structures [5,6] and spiral wire structures [2], in addition to irregular texture-type fiber/wire materials made of short straight fibers/wires [1,7,8]. Wire-woven porous material is a type of typical periodic fiber network, e.g., WBK (Wire-woven Bulk Kagome) [9], WBD (Wire-woven Bulk Diamond) [10], and WBC(Wire-woven Bulk Cross) [11]. These studies also focused on the effects of the processing conditions and network structure on mechanical response characteristics under various porosities. Zhou [12] concluded that the porosity and sintering parameters have a significant effect on the uniaxial tensile properties of the PMFSS (Porous Metal Fiber Sintered Sheet), e.g., low porosity corresponds to high tensile strength, and the tensile strength increases with increasing sintering temperature, but it decreases with increasing sintering time. Liu [3] and Tan [2] not only came to similar conclusions as Zhou on the influence of porosities on the uniaxial tensile properties, but they also investigated the effect of structural features on mechanical behaviors and properties.

Metallic wires have several merits as a raw material for porous metals. Specifically, the wires are easy to obtain, and they have a high strength, a low price, and low defects [13]. The present work involves a self-developed rotary multi-cutter device to fabricate new raw materials for use as porous metal materials. A large number of uniaxial tensile tests will be performed to investigate the effects of the sintering parameters, porosities, wire diameters, and sampling directions on the tensile properties of the PTWMs. In addition, this research also addresses the metallurgicalbonding types and the pore structures of the stainless steel PTWMs.

## 2. Results and Discussions

### 2.1. Characteristics of the Twisted Short Wires

**Figure 1:**
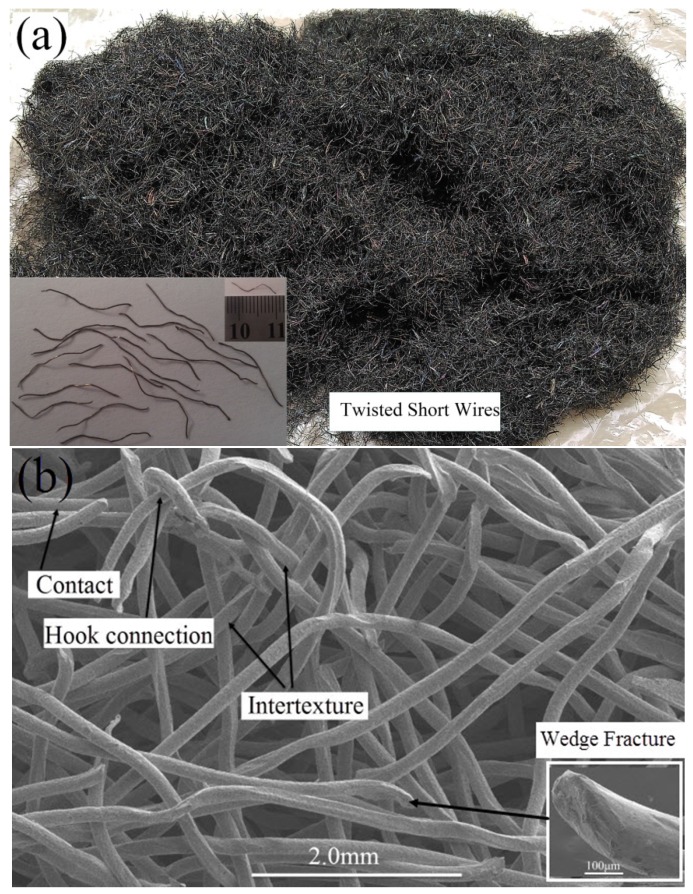
(**a**) Appearance; (**b**) SEM image of twisted short wires fabricated by the self-developed rotary multi-cutter device under natural stacking.

The single stainless steel twisted wires are in a bending-torsion deformation (Figure 1a) and present wedge-shaped fractures (Figure 1b). As shown in Figure 1b, more interweaved and intertwined conjunctive joints are formed under natural stacking, except the basic contact connection among the twisted wires. Complex connections among twisted wires may become large numbers of sintering necks after being sintered.

### 2.2. Structural Characterization of the Stainless Steel PTWMs

**Figure 2:**
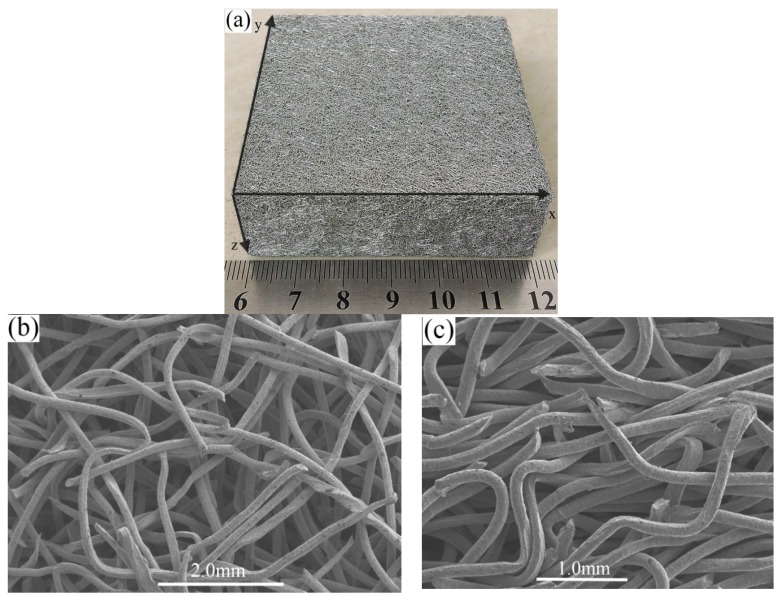
Images of the stainless steel PTSWMs: (**a**) macroscopic appearance and SEM images with 69.5% porosity; (**b**) in-plane (*x-y* plane) direction; (**c**) through-thickness direction (*x-z* plane).

Figure 2a shows the microscopic appearance of the as-prepared stainless steel PTWMs with a porosity of 69.5%. The twisted wires and pore structure are distributed randomly. Figure 2b,c clearly show the porous structure of the prepared sample with 69.5% porosity in the in-plane (*x-y* plane) and through-thickness (*x-z* plane) directions, respectively. It is notable that the stainless steel PTWM exhibits a three-dimensional composite intertexture structure. The pore structures in the in-plane direction are very different from those of the through-thickness direction. The contacted and interwoven wires form *x-y* plane pore structures. Pore skeletons basically maintain the original bending-torsion compound deformation of twisted wires and some skeletons show signs of being compressed straight (Figure 2b). The spatial structure in the through-thickness direction is constructed through the intertwining, inter-embedding, interweaving, and contact connections among twisted wires. The bending-torsion compound deformation degree of pore skeletons increases on the sides (Figure 2c). The pore structures of the PTWMs are different from the three-dimensional reticulated structure formed by straight fibers [3,8,14] and the structures of entangled materials [2,4,5,10].

**Figure 3:**
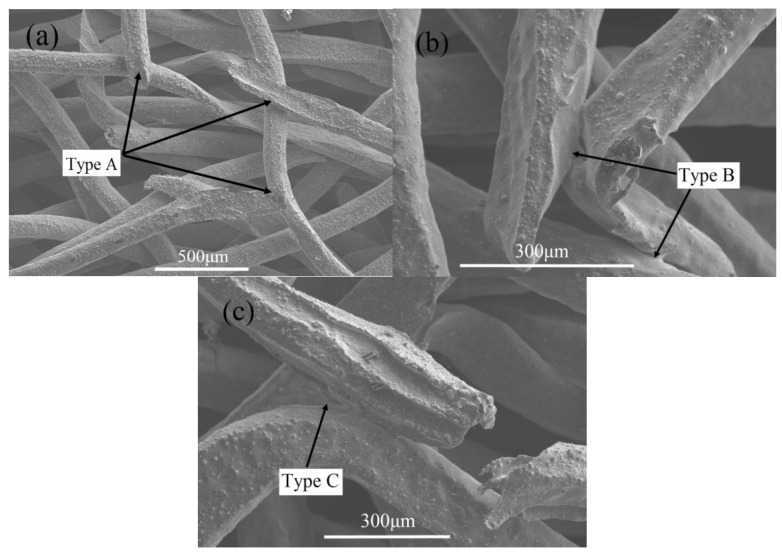
SEM images of bonging types of the stainless steel PTWMs. (**a**) A-type sintering joints; (**b**) B-type sintering joints; (**c**) C-type sintering joints.

Figure 3 shows the primary sintering joints in the stainless steel PTWMs. A-type sintering joints are formed between twisted wires by wire-to-wire surface contact, as shown in Figure 3a. Figure 3b shows the B-type metallurgical bonding among wedge-shaped fractures, curved segments, and straight segments of twisted wire after being sintered, which exhibits a triangle pore structure. Wedge-shaped fracture, curved segment and straight segment of twisted wire are sintered to bond together, which forms the C-type sintering neck (Figure 3c). Compared with the joint points, as mentioned in [3,10,12,15], twisted wires with bending-torsion deformation and wedge-shaped fractures have enriched the metallurgical bonding types of pore skeletons.

### 2.3. Tensile Properties of the Stainless Steel PTWMs

#### 2.3.1. Two Types of Typical Uniaxial Tension Failure Properties in the Stainless Wire PTWMs

**Figure 4:**
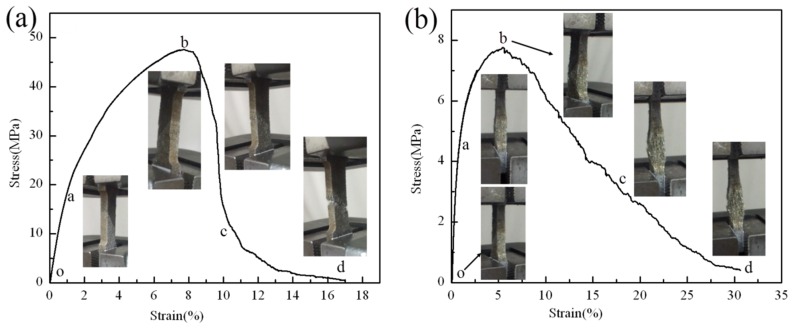
Two types of typical uniaxial tension stress-strain curves and failure process of the stainless wire PTWMs (**a**) a 45° angle fracture mode; (**b**) an auxetic failure mode.

Figure 4a,b show the uniaxial tension failure properties of the PTWMs with 61.6% and 69.4% porosity when sintered at 1330 °C and 1130 °C for 90 min, respectively. The uniaxial tension fracture processes of the PTWMs come down to the following process: Their tensile processes initially exhibit short-term elastic deformation (oa) and then quickly enter into yielding and start plastic deformation; the stress then quickly increases until reaching its maximum value with further plastic deformation (ab). Upon reaching the maximum value, macro cracks appear. After this point, the stress begins to decrease slowly with the expansion of the crack until complete failure (bd) is reached. There are no obvious yield stages throughout the stress-strain curves. The changing processes of the uniaxial tension are slow from maximum stress to complete failure. Although the stress-strain curve shown in Figure 4a is similar to that shown in Figure 4b, their macro tension failure properties are very different. Figure 4a shows a 45° angle fracture mode. Figure 4b exhibits an auxetic failure mode (as the material is stretched, it expands, rather than compresses, along some axis perpendicular to the tension [16]; this phenomenon, researchers refer to as “auxeticity”).

**Figure 5:**
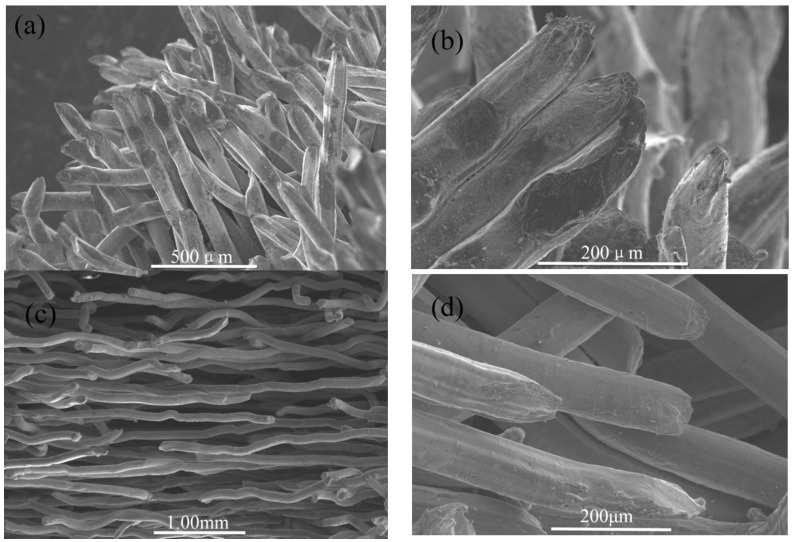
SEM images of the failed wires in the PTWMs. (**a**) and (**b**) 45° angle fracture mode; (**c**) and (**d**) auxetic failure mode.

For both samples, the stress-strain curves exhibit short-term linear elastic deformation during the initial oa stage. The stress-strain relation conforms to Hooke’s law [3]. Macro tensile diagrams of the PTWMs show almost no obvious deformation. The ab stage of the tensile curve deviates from line oa, in which the stress exceeds the elastic limits. The PTWMs enter into a complex plastic deformation that is non-uniform because of the irregular pore structure, the different lengths of the pore skeleton and the different orientation of the pore skeleton relative to the tensile loading direction. The beginning of the ab stage occurs when the local yielding of some preferable pore structure or skeleton begins, which is when the stress concentration first reaches its yield strength. The local yielding was continuously passed along, leading to tensile stress redistribution in the porous structure, resulting in the beginning of plastic deformation from the weaknesses of the pore structure or skeleton. Deformation hardening that occurs with the plastic deformation enhances PTWM deformation resistance, which results in continuous increases in stress up to the maximum stress. Macro cracks appear under maximum stress point b. After this point, the stress begins to decrease slowly with the expansion of the crack until reaching complete failure (bd). Figure 4a shows that the uniaxial tensile fracture process of the PTWMs is similar to that of the porous copper fiber materials [12]. The angle of the fracture direction in the PTWMs is approximately 45° relative to the tensile load direction. This result is consistent with the direction of maximum shear stress in the plastic flow failure principle for metal materials [12]. We do not observe any macroscopic necking phenomenon (Figure 4a), But all twisted short wires go through necking deformation to form ductile fractures (Figure 5a,b). Figure 4b shows that the lateral dimension of the sample gradually grows larger, rather than smaller, with the increasing tensile stress, which exhibits auxetic characteristics just like negative Poisson’s ratio material. The pore structure is fluffy and tears little by little during the fracture process, and it becomes looser. The sample appears to have no macroscopic fractures on the whole. Figure 5c shows that some twisted wires in the array are broken while others are stretched along the tensile direction. The fractured wires also go through necking as shown in Figure 5d. Different deformation processes lead to different stress-stain curves as in the bd section shown in Figure 4a,b.

#### 2.3.2. Effect of the Sintering Parameters on the Uniaxial Tensile Behavior

**Figure 6:**
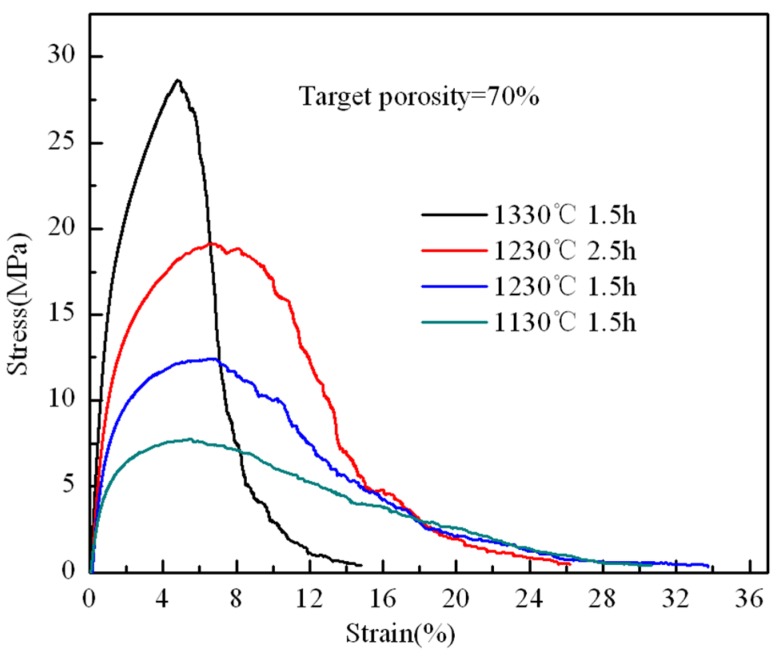
Uniaxial tensile stress-strain curves of the stainless steel PTWMs with 70% target porosity under the different sintering process.

Figure 6 shows the stress-strain curves of the stainless steel PTWMs with 70% target porosity under a different sintering process. Table 1 summarizes the sintering parameters, porosities and tensile properties of the PTWMs with 70% target porosity. The sintering temperature and time have a significant effect on the uniaxial tensile properties [3,7,12,17].

**Table 1:** Summary of the tensile properties of the PTWMs with different sintering parameters.

Sintering Parameters	Target Porosity (%)	Porosity after Sintering (%)	Ultimate Tensile Strength (MPa)	Elongation at F-max (%)	Elongation at Total Failure (%)
90 μm 1130 °C ×1.5 h	70	69.4	7.7	5.4	30.6
90 μm 1230 °C × 1.5 h	70	69.1	12.4	6.6	33.7
90 μm 1230 °C × 2.5 h	70	69.3	19.1	6.8	26.5
90 μm 1330 °C × 1.5 h	70	69.5	28.6	4.8	14.8

##### Sintering Temperature

Three different PTWM samples were obtained by sintering the twisted wires at 1130 °C, 1230 °C, and 1330°C for 90 min. Their target porosity is 70%, but the porosities after sintering are 69.4%, 69.1%, and 69.5%, respectively. The tensile stress-strain curves of the PTWMs have similar trends as those illustrated in the previous section. The sintering temperatures have a large effect on the ultimate tensile strength, elongation at the maximum stress and that of total failure, as shown in Figure 6 and Table 1.

The ultimate tensile strengths of the PTWM samples sintered at 1130 °C, 1230 °C, and 1330 °C for 90 min are 7.7 MPa, 12.4 MPa, and 28.6 MPa, respectively. It is clear that the tensile strength at 1230 °C is 1.6 times of that under 1130 °C, and the tensile strength at 1330 °C is 2.3 times of that under 1230 °C. The PTWMs with the highest tensile strength are obtained with the highest sintering temperatures. Therefore, the ultimate tensile strength has been influenced by the sintering temperature and is more sensitive to high-temperature sintering. The influence of the sintering temperature on the elongation of the maximum stress and total failure is not similar to that of the ultimate tensile strength. When the sintering temperature increases from 1130 °C to 1230 °C, the elongation at the maximum stress also increases from 5.45% to 6.6%. However, when the temperature continues to rise to 1330 °C, the elongation drops to 4.8% instead. The elongation of the total failures at 1130 °C and 1230 °C are 30.6% and 33.7%, respectively. However, the elongation of the total failure at 1330 °C is approximately half the elongation of the two former ones, namely 14.8%. The elongation of the maximum stress and total failure first increase and then decrease with the temperature increases from 1130 °C to 1330 °C.

During the vacuum sintering process, sintering points, or necks, are formed as a result of material migration between wires or wire bundle interconnection points, and then the metallurgical bonding of wires occurs as shown in Figure 3. The sintering joints or necks formed among wires grow quickly and become coarser with the increasing sintering temperature. In addition, the higher temperature is useful for increasing the atomic migration distance, which increases the probability of metallurgical bonding among the neighbor wires and the metallurgical bonding area per unit volume. It is without doubt that the metallurgical bonding joints, or necks, in the PTWMs play an important role in terms of their mechanical properties and the structural effect [5]. Thus, the increase in the sintering temperature will enhance the ultimate tensile strength. The growth or increase of metallurgical bonding joints or necks also strengthens the deformation resistance of the PTWMs and leads to difficulty in the deformation passing through the porous structure. The delay in crack propagation reduces the speed of the sample’s total failure and increases the failure elongation. Therefore, the ultimate tensile strength is greater, but the failure elongation decreases with the increasing sintering temperature from 1130 °C to 1330 °C.

##### Sintering Time

Figure 6 shows the stress-strain curves of the PTWMs that were sintered at 1230 °C for 90 min and 150 min. The target porosity is 70%, but the actual porosities are 69.1% and 69.2%, respectively. In comparison with the tensile strength of 12.4 MPa and the total failure elongation of 33.7% for the sample that was sintered for 90 min, the tensile strength of the sample sintered for 150 min increases to 19.1 MPa, and its total failure elongation drops to 26.5%. However, these two samples have similar elongation values at the maximum stress. Thus, the ultimate tensile stress and its elongation increase by a small amount; the total failure elongation decreases instead with the increasing sintering time. We attribute these results to the following findings: A longer sintering time makes the metallic atoms diffuse deep enough to lead to the growth of the sintering necks or the formation of more sintering necks, which also increases the fracture deformation resistance.

#### 2.3.3. Effect of the Wire Diameters on the Uniaxial Tensile Properties

**Figure 7:**
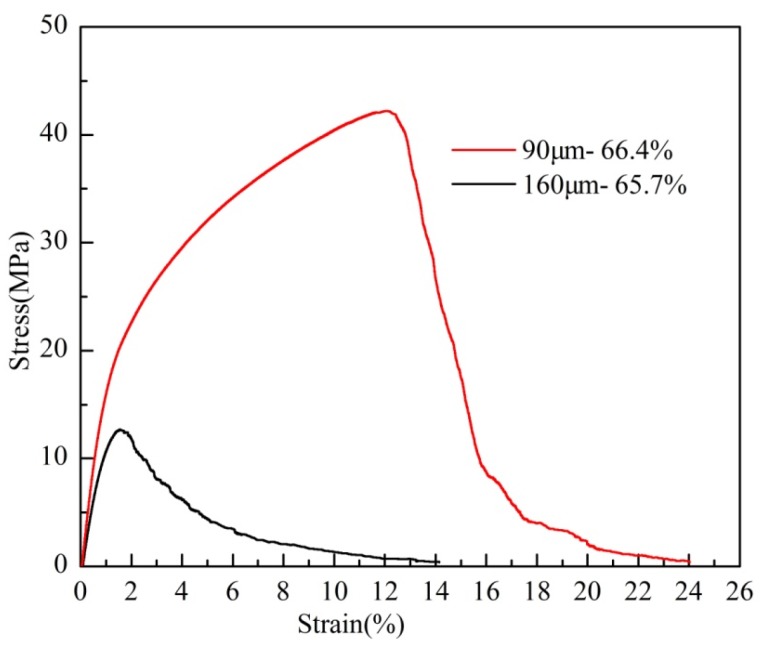
Uniaxial tensile stress-strain curves of the PTWMs with different wire diameters.

Figure 7 shows the stress-strain curves of the PTWMs with 66.4% and 65.7% porosities by using the twisted wires with diameters of 90 μm and 160 μm, respectively, which were sintered at 1330 °C for 90 min; this finding is consistent with the tensile stress-strain curve as shown in Figure 4a. The tensile strength, the elongation at the maximum stress and the total failure elongation of the PTWMs with a diameter of 90 μm are 42.2 MPa, 12% and 24%, respectively; the values for PTWMs with a diameter of 160 μm are 12.0 MPa, 1.2% and 14.1%, respectively. The tensile strength, the elongation at the maximum stress and the total failure elongation of the PTWMs with a diameter of 90 μm are 3.5, 9.6 and two times those of the PTWMs with a diameter of 160 μm. The PTWMs with fine wires contain more twisted wires per unit volume under the same porosity. The pore structure of the cross section perpendicular to the loading direction is denser. When the PTWMs with fine wires are stretched, more twisted wires and pore structures participate in the tensile process. Therefore, the tensile strength and the failure strain of the PTWMs with fine wires are higher than that of the PTWMs with coarse wires. The diameter of the twisted wire has a clear effect on the tensile properties of the PTWMs.

#### 2.3.4. Uniaxial Tensile Behavior with Different Porosities

**Figure 8:**
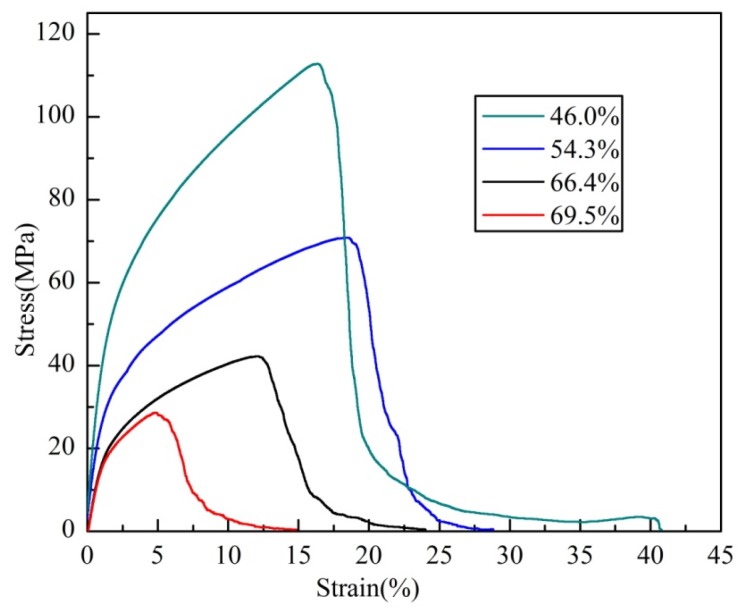
Uniaxial tensile stress-strain curves of the PTWMs with different porosities.

Figure 8 shows the uniaxial tensile stress-strain curves with different porosities which is consistent with the tensile stress-strain curve shown in Figure 4a. However, the slopes and extents of the curves are different. The tensile properties of the PTWMs with different porosities are summarized in Table 2. The PTWMs exhibit the biggest elastic slope, the highest ultimate tensile strength and the longest failure elongation with the lowest porosity.

**Table 2:** Summary of the tensile properties of the PTWMs with different porosities.

Porosity after Sintering (%)	Sintering Parameters	Ultimate Tensile Strength (MPa)	Elongation at F-max (%)	Elongation at Total Failure (%)
46.0	90μm 1330 °C × 1.5 h	112.7	16.3	40.7
54.4	90μm 1330 °C × 1.5 h	70.8	18.3	28.8
66.4	90μm 1330 °C × 1.5 h	42.2	12.0	24.0
69.5	90μm 1330 °C × 1.5 h	28.6	4.8	14.8

The ultimate tensile strength, the elongation at the maximum stress and the total failure elongation of the PTWMs drop from 112.7 MPa, 16.3% and 40.7% to 28.6 MPa, 4.8% and 14.8% when the porosity increases from 46% to 70.1%, respectively. As expected, the tensile properties of the PTWMs strongly depend on the porosities. The lower porosity contains more twisted wires per unit volume and leads to a more compact pore structure, which facilitates the formation of more conjunctive joints among wires which, then, subsequently turn into more metallurgical bonding joints or necks after sintering. The above metallurgical bonding can be considered with firmer welding among the wires so that the PTWMs have a higher tensile strength. There were lower porosity results in the denser pore structure, which leads more twisted wires and pore structures to participate in the tensile process. The tensile failure process must overcome more twisted wires and pore structures to further destruction. This extra step delays the complete failure process. Therefore, unlike the sintering parameter effects on the failure elongation, the tensile strength and the failure elongation both increase with the decreasing porosity.

#### 2.3.5. Effect of the Sampling Direction on the Uniaxial Tensile Properties

**Figure 9:**
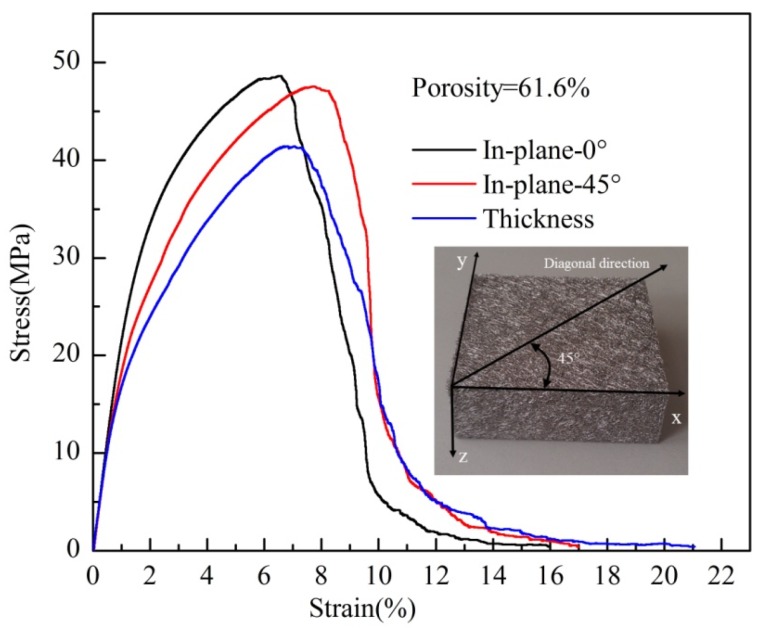
Uniaxial tensile stress-strain curves of the PTWMs in the in-plane and through-thickness direction.

Figure 9 shows the tensile stress-strain curves of the PTWMs with 61.6% porosity when sintered at 1330 °C for 90 min in the in-plane and through-thickness directions, which are consistent with the tensile stress-strain curve as shown in Figure 7a. The tensile samples were cut in the in-plane 0° direction (parallel to the *x* or *y* direction), the in-plane 45° direction (the diagonal direction) and the through-thickness direction (along the *z* direction in the *z-x* plane), as shown in Figure 9. Their tensile properties are summarized in Table 3. The stress-strain curves of the three samples appear to have some overlap during the elastic deformation stage and subsequently exhibit varying degrees of deviation. The tensile strength, elongation at the maximum stress and the total failure elongation of the in-plane 0° sample are 48.6 MPa, 6.5% and 15.9%, respectively. The values for the in-plane 45° sample are 47.5 MPa, 7.7% and 16.9%, respectively. These data show that the tensile strength and the elongation of the PTWMs within the in-plane direction at 0° and 45° differ very little. The tensile strength, the elongation at the maximum stress, and the total failure elongation for the through-thickness direction are 41.4 MPa, 6.6% and 21%, respectively. In comparison with the in-plane 0° sample, the tensile strength in the through-thickness direction is reduced by 7.1 MPa, and the total failure elongation increases 50%. These results occur because the bending degree of twisted wires is larger than that of the in-plane direction in the through-thickness direction. The tensile stress first strengthens the twisted wires, then makes random pore structures form a consistent linear column along the tensile loading direction. The above process delays the complete failure course. Therefore, the tensile strength decreases and the total failure elongation increases, instead. The tensile properties are anisotropic between the in-plane and through-thickness directions, which may be attributed to the different porous structures in these directions as shown in Figure 2.

**Table 3:** Summary of the tensile properties of the PTWMs with different sampling direction.

Sintering Parameters	Porosity after Sintering (%)	Sampling Direction	Ultimate Tensile strength (MPa)	Elongation at F-max (%)	Elongation at Total Failure (%)
90μm 1330 °C × 1.5 h	61.6	in-plane (0°)	48.6	6.5	15.9
90μm 1330 °C × 1.5 h	61.6	in-plane (45°)	47.5	7.7	16.9
90μm 1330 °C × 1.5 h	61.6	through-thickness	41.4	6.6	21.0

## 3. Experimental Procedures

The experimental procedures consisted of three parts, namely preparation of stainless steel twisted short wires, fabrication of stainless steel porous twisted wire materials (PTWMs) and tensile testing.

### 3.1. Preparation of the Stainless Steel Twisted Short Wires

**Figure 10:**
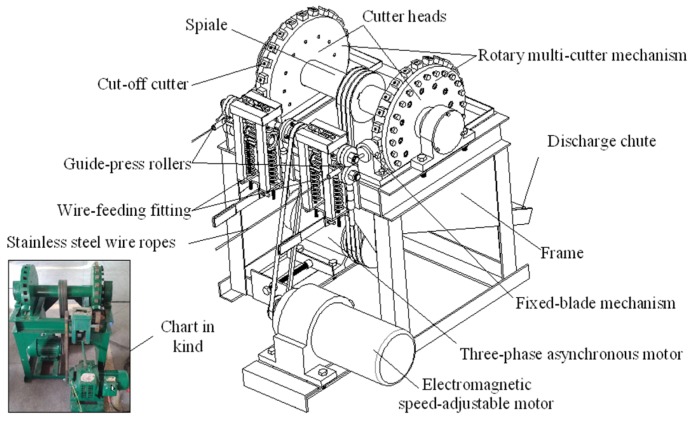
The rotary multi-cutter device to cut off steel wire ropes.

The stainless steel twisted short wires were pre-formed by using self-developed rotary multi-cutters (Figure 10) to cut stainless steel wire ropes into segments with lengths ranging from 10–15 mm (Figure 1). Stainless steel ropes (06Cr19Ni10) that measured 90 μm and 160 μm in (wire) diameter were used as the basic materials.

As shown in Figure 10, the rotary multi-cutter device primarily consists of a frame, a wire-feeding fitting, a rotary multi-cutter mechanism, a fixed-blade mechanism, and a control drive unit. The lower roller is embedded in the ring groove of the upper roller, which forms a pair of opposite-rotating wire-feeding friction rollers called guide-press rollers. There is a gap between two work rollers that can be adjusted by radially moving the lower roller, between which fibers/wires/steel wire ropes are guided. Two pairs of guide-press rollers are distributed symmetrically on the left and right sides of the frame. The above assembly forms the wire-feeding fitting. The rotary multi-cutter mechanism includes cut-off cutters, cutter heads, and a shaft. Two cutter heads are distributed symmetrically on both sides of the shaft. Each cutter head has 24 pieces of cut-off cutters that are evenly distributed along its circular form. The fixed-blade mechanism is located between the wire-feeding fitting and the rotary multi-cutter mechanism, and it consists of a fixed-blade holder and a die cutter. The fixed-blade die cutter features a through-hole in its axial direction for fibers/wires/steel wire ropes.

When the apparatus is working, steel wire ropes are placed in the gap between the upper and lower roller. The wires are fed forward continuously because of the friction action between the guide-press rollers and the steel wire ropes, while an electromagnetic speed-adjustable motor drives the guide-press rollers that were rotating through the belt pulley (Figure 10). The extension of steel wire ropes is variable with the rotation speed of the guide-press rollers, which are varied by controlling an electromagnetic speed-adjustable motor. A three-phase asynchronous motor drives the shaft that is rotating through the action of a belt pulley. Two cutter heads, which are installed on the shaft, also rotates to drive the 48 pieces of cut-off cutters that are mounted on the heads. The rotary cut-off cutters cooperate with the fixed blade to cut off the steel wire ropes continuously. The steel wire ropes will automatically spread out to form short twisted wires.

### 3.2. Fabrication of the Stainless Steel PTWMs

The processing procedure for stainless steel PTWMs was divided into the following two steps: mold-pressing and vacuum sintering.

**Figure 11:**
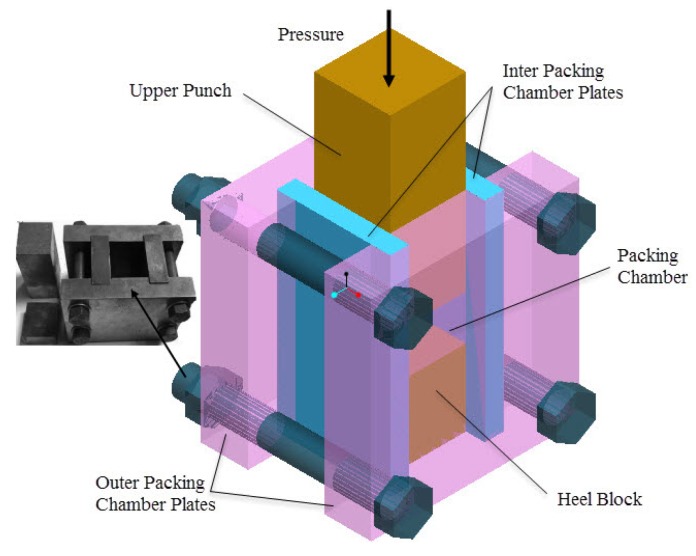
Mold-pressing equipment of twisted short wires.

At first, the as-prepared twisted wires in an entangled state were placed inside a specially designed packing chamber of the mold pressing equipment as shown in Figure 11, then different pressures were applied to the upper punch to compact the twisted short wires. In this way, preforms of the PTWMs were obtained. Next, the compacted samples were sintered in a vacuum furnace (WHS-20 vacuum sintering furnace). A stage heating method was used in the experiment. The heating rate was kept at 10 °C·min^−1^ when the temperature was below 800 °C, and it was reduced to 6 °C·min^−1^ when the temperature was above 800 °C. The sintering temperature was maintained between 1130 °C and 1330 °C. The sintering time was either 90 min or 150 min. The samples were naturally-cooled to room temperature in the chamber of a vacuum furnace. During the sintering process, the vacuum device (the vacuum degree was approximately 1 × 10^−2^ Pa) had been opened to prevent the stainless steel PTWMs from oxidation until the temperature dropped to 100 °C.

### 3.3. Characterization and Tensile Test of the PTWMs

The average porosities of stainless steel PTWMs were calculated by using the quality-volume method that was formulated as:

(1) $$P\left(\%\right)=\left(1-\frac{M}{\rho V}\right)\times 100$$

where P is the average porosity of stainless steel PTWMs, M is the mass of porous material (g), V is the volume of porous sample (*cm*^3^), and ρ is the density of the solid 304 stainless steel (*g*/*cm*^3^).

A Hitachi (Tokyo, Japan) S-3700N scanning electron microscope (SEM) was used to observe the microscopic structure of the stainless steel PTWMs. The tensile samples were prepared in a dog-bone shape. The gauge sections were 25 mm long, 10 mm wide, and 5 mm thick. A length of approximately 10 mm at each end of the tensile samples was infiltrated with epoxy resin. After the resin had cured, these infiltrated sections were held in grips, and testing was performed with an electronic universal mechanical testing machine (No: AG-100NX, Shimadzu, Kyoto, Japan) at a constant crosshead speed of 0.6 mm·min^−1^.

## 4. Conclusions

(1) The self-developed rotary multi-cutter device is helpful in the bulk production of short fibers/wires, which makes it possible to mass produce porous metal fiber network materials. This cutting device quickly and efficiently cuts stainless steel wire ropes into segments to fabricate the twisted wires that exhibit bending-torsion deformation and wedge-shaped fractures. The stainless steel PTWMs with spatial composite intertexture structures are produced by compaction and the subsequent vacuum solid-phase sintering of twisted wires.
(2) The stainless wire PTWMs show two types of typical uniaxial tensile failure modes, *i.e.*, a 45° angle fracture failure and an auxetic failure mode. The uniaxial tension fracture process can be described as follows: The tensile process initially exhibits short-term elastic deformation, and then it quickly enters into yielding and starts plastic deformation; the stress then quickly increases until reaching its maximum value with further plastic deformation. Upon reaching the maximum value, macro cracks appear. After that, the stress will begin to decrease slowly with the expansion of the crack until reaching complete failures. There is no obvious yield stage throughout the stress-strain curve. The changing process of uniaxial tension is slow from the maximum stress to the complete failure.
(3) The sintering temperature and sintering time have large effects on the uniaxial tensile properties of the PTWMs. The ultimate tensile strength of the PTWMs with 70% target porosity increases from 7.7 MPa to 28.6 MPa with increasing the sintering temperature from 1130 °C to 1330 °C and the total failure elongation decreases from 30.6% to 14.8%. The tensile strength increases from 12.4 MPa to 19.1 MPa and the total failure elongation drops from 33.7% to 26.5% with increasing the sintering time from 90 min to 150 min. The influence of the sintering temperature on the tensile properties of the PTWMs is greater than that of the sintering time.
(4) Unlike the sintering parameters that influence the failure elongation, the tensile strength and the failure elongation of the PTWMs both increase with the porosity decrease and strongly depend on porosities. The tensile strength of the PTWMs increases from 28.9 MPa to 112.7 MPa and the failure elongation increased from 14.8% to 40.7% with decreasing porosity from 69.5% to 46.0%.
(5) The tensile strength, the elongation at the maximum stress and the total failure elongation of the PTWMs with a diameter of 90 μm are 3.52, 9.66 and 2.08 times those of the PTWMs with a diameter of 160 μm. The tensile strength and the failure strain of the PTWMs with fine wires are higher than that of the PTWMs with coarse wires. The diameter of the twisted wire has a clear effect on the tensile properties of the PTWMs.
(6) The tensile strength and the elongation of the PTWMs within the in-plane direction at 0° and 45° differ very little. The tensile properties are anisotropic between the in-plane and through-thickness directions, which may be attributed to the different porous structures of the two directions.
